# “I WON’T GO THROUGH THAT OR TAKE MY FAMILY THROUGH THAT”: FAMILY RELATIONSHIPS AND KIDNEY DISEASE DECISION-MAKING

**DOI:** 10.1093/geroni/igae098.3619

**Published:** 2024-12-31

**Authors:** Tyrone Hamler, Asia Cutforth, Emily Miller, Kari O’Donnell

**Affiliations:** University of Denver, Denver, Colorado, United States; University of Denver, Denver, Colorado, United States; Case Western Reserve University, Cleveland, Ohio, United States; MetroHealth, Cleveland, Ohio, United States

## Abstract

Chronic kidney disease (CKD) uniquely impacts older Black Americans, who are nearly four times more likely to develop kidney failure than older White Americans and more likely to have family members also diagnosed with CKD. Utilizing a phenomenological approach, this study explored participants’ perceptions of how family members’ prior experiences with CKD influenced treatment-related decision-making. This study aimed to (1) describe how older Black adults viewed the experiences of family and friends previously diagnosed with CKD and (2) to understand how these social complexities informed their own choices for future CKD care. A reflective, thematic content analysis was conducted to identify patterns across participant responses (N=52). Participants were pre-dialysis, diagnosed with stage four or five chronic kidney disease, and were receiving outpatient nephrology care at a large, urban midwestern hospital. Three primary themes emerged: (1) Decision-Making Factors, (2) Health Inequities, and (3) Continuity of Care. These three interconnected themes centered on factors that influenced why and how decisions related to chronic kidney disease were made and how racial identity and prior family experiences with CKD influenced these choices. Over the next 40 years, the population of Black Americans aged ≥ 65 years will nearly triple, and CKD will continue to be a major public health concern. Addressing the complex support requirements and burdens of CKD care is critical for reshaping the negative and disorienting experiences of older Black Americans and reducing future decision-making conflicts.

